# Correction: Novel aerosol treatment of airway hyper-reactivity and inflammation in a murine model of asthma with a soluble epoxide hydrolase inhibitor

**DOI:** 10.1371/journal.pone.0307533

**Published:** 2024-07-17

**Authors:** Chuanzhen Zhang, Wei Li, Xiyuan Li, Debin Wan, Savannah Mack, Jingjing Zhang, Karen Wagner, Chang Wang, Bowen Tan, Jason Chen, Ching-Wen Wu, Kaori Tsuji, Minoru Takeuchi, Ziping Chen, Bruce D. Hammock, Kent E. Pinkerton, Jun Yang

The following information is missing from the Funding statement: This study was supported by the Cultivating Fund for National Natural Science Foundation of China of Shandong Provincial Qianfoshan Hospital (QYPY2019NSFC0603).
