# Supplementary material for: Novel aerosol treatment of airway hyper-reactivity and inflammation in a murine model of asthma with a soluble epoxide hydrolase inhibitor
Source: PLoS One. 2022 Apr 20;17(4):e0266608. doi: 10.1371/journal.pone.0266608 (PMC9020733; doi:10.1371/journal.pone.0266608)
Supplement: S1 Table — (DOCX) [file pone.0266608.s001.docx]

**S1 Table. Semi-quantitative scoring rubric for the severity of inflammation in lung tissues.**

| **Score** | **Alveolitis** | **Bronchiolitis** | **Perivasculitis** | **Pleuritis** |
| --- | --- | --- | --- | --- |
| 0 | Normal. Thin alveolar walls, with rare macrophages in the lumen. No inflammatory cells. | Normal 1 cell-layer thick epithelium, smooth muscle and submucosal layers. | Normal vascular endothelium. | Rare/no cells at the pleura. |
| 1 | Similar to 0 score, with more free macrophages in the lumen. No polymorphonuclear cells (PMNs). | Slight influx of Macrophages and/or monocytes into the submucosa, without PMNs. | Slight influx of a few macrophages and/or monocytes into this region, without PMNs. All the connective tissue is almost visible. | Slightly increased cellularity, without PMNs. |
| 2 | Atypical cellularity in the alveolar walls, and/or the majority of spaces in alveolar lumen still clear of free cells. Macrophages, monocytes, and/or PMNs are over-represented. | Mildly thickened the airway, with moderate influx of PMNs (<15% of infiltrated cells) and/or phagocytes into the submucosa. | Much of the connective tissue is infiltrated with moderate influx of PMNs and/or phagocytes, but still visible. | Moderately increased cellularity, with PMNs and/or phagocytes. |
| 3 | Alveolar walls thicken. Obvious influx of phagocytes and/or PMNs into the lumen forms large cellular agglomerates occupying much of the lumen airspace. | Markedly thickened airway, with obvious influx of inflammatory cells into the submucosa. A high percentage of PMNs is unnecessary. | Much of the tissue is markedly infiltrated with mixed cells so as to be obscured. A high percentage of PMNs is unnecessary. | Severe influx of cells into the pleura. A high percentage of PMNs may be present, along with foamy macrophages. |

Note: Reproduced and adapted from “Long-Term Sequelae of Smoking and Cessation in Spontaneously Hypertensive Rats.” by Wu et al. [41] with the authors’ permission.
