# Supplementary material for: Novel aerosol treatment of airway hyper-reactivity and inflammation in a murine model of asthma with a soluble epoxide hydrolase inhibitor
Source: PLoS One. 2022 Apr 20;17(4):e0266608. doi: 10.1371/journal.pone.0266608 (PMC9020733; doi:10.1371/journal.pone.0266608)
Supplement: S2 Table — (DOCX) [file pone.0266608.s002.docx]

**S2 Table. Semi-quantitative scoring rubric for the severity of airway constriction.**

| **Score** | **Brief description** |
| --- | --- |
| 0 | Airways exhibit little to no signs of constriction and appear to be healthy. |
| 1 | Airways exhibit signs of constriction such as frills or convolutions along the edges but are not tightly constricted or in contact with one another. |
| 2 | Airways have frills or convolutions that are constricted and are in contact with one another, but they have a large amount of open airway space. |
| 3 | Airways are severely contracted in that they have constricted entirely and become extremely dense, with little to no open airway space. |
