# Supplementary material for: Novel aerosol treatment of airway hyper-reactivity and inflammation in a murine model of asthma with a soluble epoxide hydrolase inhibitor
Source: PLoS One. 2022 Apr 20;17(4):e0266608. doi: 10.1371/journal.pone.0266608 (PMC9020733; doi:10.1371/journal.pone.0266608)
Supplement: S3 Table — (DOCX) [file pone.0266608.s003.docx]

**S3 Table. Concentrations of lipid mediators in BALF of mice inhaling OVA + TPPU (2 h) versus OVA alone.**

| **Lipid mediator** | **OVA (nmol/L) (Mean ± SEM)** | **OVA + TPPU (2 h) (nmol/L) (Mean ± SEM)** | **FC** | ***p-*value** |
| --- | --- | --- | --- | --- |
| 6-keto-PGF1a | 18.84 ± 5.06 | 4.97 ± 1.06 | 0.26 | 0.02* |
| TXB2 | 0.67 ± 0.30 | 0.18 ± 0.06 | 0.27 | 0.11 |
| PGF2a | 2.25 ± 1.33 | 0.27 ± 0.10 | 0.12 | 0.13 |
| PGE2 | 3.05 ± 1.61 | 0.38 ± 0.14 | 0.12 | 0.10 |
| PGD2 | 1.38 ± 0.67 | 0.20 ± 0.05 | 0.14 | 0.08 |
| LTB4 | 0.14 ± 0.04 | 0.06 ± 0.02 | 0.41 | 0.06 |
| 5-HETE | 2.03 ± 0.76 | 0.24 ± 0.10 | 0.12 | 0.03* |
| 5-HEPE | 0.44 ± 0.16 | 0.10 ± 0.01 | 0.22 | 0.04* |
| 12-HETE | 62.07 ± 29.65 | 26.68 ± 13.68 | 0.43 | 0.28 |
| 12-oxo-ETE | 68.25 ± 40.67 | 61.08 ± 21.90 | 0.89 | 0.87 |
| 12-HEPE | 6.70 ± 3.92 | 6.06 ± 2.18 | 0.90 | 0.88 |
| 9-HODE | 5.01 ± 2.11 | 0.96 ± 0.29 | 0.19 | 0.07 |
| 9-oxo-ODE | 4.66 ± 2.50 | 0.82 ± 0.23 | 0.18 | 0.13 |
| 13-HOTrE | 0.51 ± 0.22 | 0.41 ± 0.18 | 0.80 | 0.72 |
| 15-HETE | 10.17 ± 4.12 | 4.88 ± 2.74 | 0.48 | 0.30 |
| 15-oxo-ETE | 2.93 ± 1.27 | 0.47 ± 0.20 | 0.16 | 0.07 |
| 15(S)-HETrE | 1.38 ± 0.65 | 0.86 ± 0.49 | 0.63 | 0.54 |
| 17-HDoHE | 16.12 ± 7.84 | 10.04 ± 4.50 | 0.62 | 0.50 |
| 15-HEPE | 2.49 ± 1.11 | 1.36 ± 0.66 | 0.55 | 0.39 |

**S3 Table. Concentrations of lipid mediators in BALF of mice inhaling OVA + TPPU (2 h) versus OVA alone (continued).**

| **Lipid mediator** | **OVA (nmol/L) (Mean ± SEM)** | **OVA + TPPU (2 h) (nmol/L) (Mean ± SEM)** | **FC** | ***P* value** |
| --- | --- | --- | --- | --- |
| 13-HODE | 12.62 ± 4.73 | 7.70 ± 3.46 | 0.61 | 0.42 |
| 8-HETE | 1.80 ± 0.84 | 0.61 ± 0.33 | 0.34 | 0.24 |
| LXA4 | 0.42 ± 0.18 | 0.08 ± 0.03 | 0.18 | 0.07 |
| 11,12,15-TriHETrE | 21.67 ± 7.07 | 2.66 ± 1.22 | 0.12 | 0.02* |
| 9,12,13-TriHOME | 23.34 ± 9.40 | 3.56 ± 1.45 | 0.15 | 0.05* |
| 9,10,13-TriHOME | 13.38 ± 5.67 | 1.30 ± 0.47 | 0.10 | 0.05* |
| 6-trans-LTB4 | 1.06 ± 0.60 | 0.34 ± 0.17 | 0.32 | 0.24 |
| 11-HETE | 1.29 ± 0.46 | 0.33 ± 0.11 | 0.25 | 0.06 |
| 9-HETE | 1.21 ± 0.48 | 0.20 ± 0.05 | 0.17 | 0.05* |
| EKODE | 12.66 ± 8.75 | 1.08 ± 0.42 | 0.09 | 0.18 |
| 15,16-DiHODE | 0.17 ± 0.03 | 0.07 ± 0.02 | 0.39 | 0.01* |
| 14,15-DiHETrE | 0.07 ± 0.01 | 0.03 ± 0.01 | 0.45 | 0.01* |
| 11,12-DiHETrE | 0.09 ± 0.03 | 0.02 ± 0.00 | 0.28 | 0.04* |
| 16,17-DiHDPE | 0.14 ± 0.06 | 0.10 ± 0.03 | 0.75 | 0.62 |
| 13,14-DiHDPE | 0.03 ± 0.02 | 0.02 ± 0.01 | 0.59 | 0.43 |
| 17,18-DiHETE | 0.20 ± 0.08 | 0.12 ± 0.02 | 0.57 | 0.28 |
| 12,13-DiHOME | 0.50 ± 0.17 | 0.17 ± 0.05 | 0.34 | 0.08 |
| 9,10-DiHOME | 0.32 ± 0.15 | 0.06 ± 0.03 | 0.20 | 0.10 |
| 15(16)-EpODE | 0.12 ± 0.03 | 0.09 ± 0.03 | 0.75 | 0.49 |

**S3 Table. Concentrations of lipid mediators in BALF of mice inhaling OVA + TPPU (2 h) versus OVA alone (continued).**

| **Lipid mediator** | **OVA (nmol/L) (Mean ± SEM)** | **OVA + TPPU (2 h) (nmol/L)**  **(Mean ± SEM)** | **FC** | ***P* value** |
| --- | --- | --- | --- | --- |
| 20-HETE | 1.26 ± 0.52 | 0.17 ± 0.06 | 0.13 | 0.048* |
| 14(15)-EET | 0.68 ± 0.23 | 0.32 ± 0.10 | 0.47 | 0.17 |
| 11(12)-EET | 1.01 ± 0.36 | 0.29 ± 0.08 | 0.29 | 0.07 |
| 8(9)-EET | 0.59 ± 0.25 | 0.23 ± 0.08 | 0.39 | 0.31 |
| 5(6)-EET | 8.46 ± 2.96 | 1.96 ± 0.53 | 0.23 | 0.045* |
| 19(20)-EpDPE | 0.83 ± 0.23 | 0.42 ± 0.12C | 0.50 | 0.13 |
| 12(13)-EpOME | 1.17 ± 0.40 | 0.44 ± 0.10 | 0.38 | 0.09 |
| 9(10)-EpOME | 1.22 ± 0.47 | 0.39 ± 0.08 | 0.32 | 0.09 |

* - statistically significant a *p* < 0.05.

Abbreviations: DiHDPE - dihydroxydocosapentaenoic acid; DiHETE - dihydroxyeicosatetraenoic acid; DiHETrE - dihydroxyeicosatrienoic acid; DiHODE - dihydroxyoctadecadienoic acid; DiHOME - dihydroxyoctadecamonoenoic acid; EET - epoxyeicosatrienoic acid; EKODE - epoxyketooctadecenoic acid; EpDPE - epoxydocosapentaenoic acid; EpODE - epoxyoctadecadienoic acid; EpOME - epoxyoctamonoemoic acid; ETE - eicosatrienoic acid; FC - fold change; HDoHE - hydroxydocosahexaenoic acid; HEPE - hydroxyeicosapentanoic acid; HETrE - hydroxyeicosatrienoic acid; HETE - hydroxyeicosatetraenoic acid; HODE - hydroxyoctadecadienoic acid; HOTrE - hydroxyoctadecatrienoic acid; LX - lipoxin; LT - leukotriene; ODE - octadecadienoic acid; PG - prostaglandin; SEM - standard errors of the mean; TriHETrE - trihydroxyeicosatrienoic acid; TriHOME - trihydroxyoctadecamonoenoic acid; TX - thromboxane; β - beta.
