# Supplementary figures and images for: Novel aerosol treatment of airway hyper-reactivity and inflammation in a murine model of asthma with a soluble epoxide hydrolase inhibitor
Source: PLoS One. 2022 Apr 20;17(4):e0266608. doi: 10.1371/journal.pone.0266608 (PMC9020733; doi:10.1371/journal.pone.0266608)

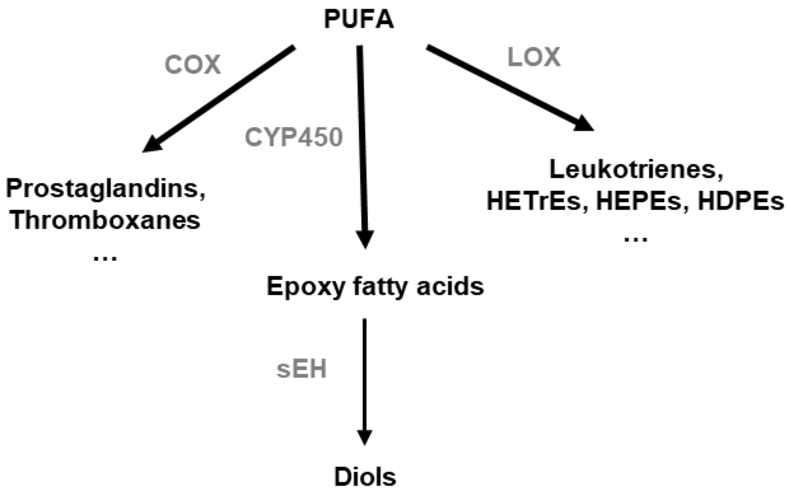

Supplement: S1 Fig — These pathways combine to produce complex products. The CYP450 pathway also produces a variety of allylic hydroxy metabolites and omega (ω) and ω-1 hydroxy metabolites, such as the pro-inflammatory vasoconstrictor 20-HETE (hydroxyeicosatetraenoic acid). Abbreviations: COX—cyclooxygenase; CYP450—cytochrome P450; HDPEs—hydroxydocosapentaenoic acids; HEPEs—hydroxyeicosapentanoic acids; HETrEs—hydroxyeicosatrienoic acids; LOX—lipoxygenase; PUFA—polyunsaturated fatty acid; sEH—soluble epoxide hydrolase. (TIF) [file pone.0266608.s004.tif]

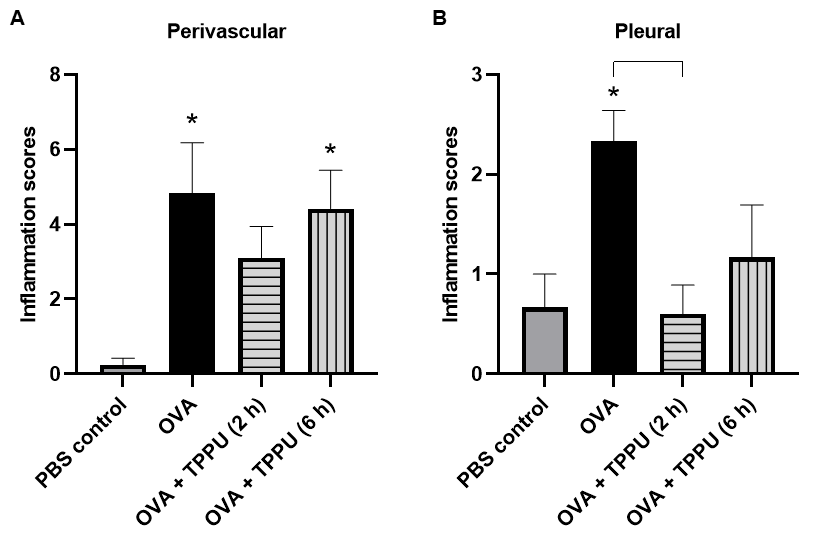

Supplement: S2 Fig — Data are shown as the mean ± standard error of the mean. An asterisk (*) denotes a significant (p < 0.05) difference compared to the PBS control, while brackets represent significant (p < 0.05) differences between the OVA and OVA + TPPU groups. A Kruskal-Wallis test and post-hoc Dunnett’s Multiple Comparisons test were used to analyze perivascular inflammation. A one-way ANOVA and post hoc Tukey’s Multiple Comparisons test were used for pleural inflammation. (TIF) [file pone.0266608.s005.tif]
